# Gonadotropin-Releasing Hormone (GnRH)/GnRH Receptors and Their Role in the Treatment of Endometriosis

**DOI:** 10.7759/cureus.38136

**Published:** 2023-04-26

**Authors:** Christina Resta, Athanasios Moustogiannis, Eirini Chatzinikita, Dimitris Malligiannis Ntalianis, Konstantinos Malligiannis Ntalianis, Anastasios Philippou, Michael Koutsilieris, Nikolaos Vlahos

**Affiliations:** 1 Department of Physiology, Medical School, National and Kapodistrian University of Athens, Athens, GRC; 2 Second Department of Obstetrics and Gynaecology, Aretaieion Hospital, University of Athens, Athens, GRC

**Keywords:** gnrh receptors, gnrh, laparoscopy, gonadotropin-releasing hormone, endometriosis

## Abstract

Endometriosis, defined as the development of endometrial tissue outside of the uterine cavity, is a common gynecological disorder. The prevalence of pelvic endometriosis approaches 6%-10% in the general female population, and in women with pain, infertility, or both, the frequency is 35%-50%. The gold standard recommended process for diagnosing endometriosis is laparoscopy, an invasive surgical procedure, with or without histologic verification. The currently available nonsurgical treatments include oral contraceptives (estrogen-progestogen preparations), progestogen preparations (containing progesterone derivatives), androgenic hormones (danazol), and gonadotropin-releasing hormone (GnRH) agonists and antagonists. Two GnRH types have been discovered in mammals, GnRH I and GnRH II. In particular, GnRH I is released by the hypothalamus; however, it can be present in various tissues and organs of the body, including neural tissue, where it exerts neuroendocrine, autocrine, and paracrine actions in the peripheral and central nervous system (CNS). Interestingly, another GnRH isoform, GnRH III, has been identified, which has 60% similarity with GnRH I from which it varies by four amino acids. This peptide has been shown to have a significant role in reproduction, specifically in gametogenesis and steroidogenesis. Further research is needed to identify innovative treatment options for endometriosis, such as the therapeutic exogenous administration of GnRH II or antagonists of the GnRH I receptor. In this review, we examined the role of GnRH in endometriosis, outlining the specific actions of GnRH and GnRH receptors (GnRHRs). The innovative use of GnRH analogs and antagonists in the treatment of endometriosis is also discussed.

## Introduction and background

Endometriosis, defined as the development of endometrial tissue outside of the uterine cavity, is a common gynecological disorder. Endometriosis has a profound impact on patients’ quality of life, causing infertility, chronic pelvic pain, dysmenorrhea, and dyspareunia, and can interfere with daily life [1]. The prevalence of pelvic endometriosis approaches 6%-10% in the general female population, and in women with pain, infertility, or both, the frequency is 35%-50% [2]. Although endometriosis is the most common disorder diagnosed in women of reproductive age, the time to diagnosis can be very long (4-11 years from onset) because of variability in symptoms and signs and confusion with other disorders [2,3].

To date, at least 1,000 papers about the epidemiology of endometriosis have been published, but its etiology remains enigmatic. Several theories have been proposed about the pathogenesis of this condition. Retrograde menstruation and growth factors, which are synthesized locally in the peritoneal cavity, are assumed to enhance the development, establishment, and maintenance of endometriotic lesions. In addition, genetic, hormonal, and immunological factors almost certainly have a role in the emergence of endometriosis symptoms. Environmental pollutants, such as bisphenol A or phthalates, have also been implicated in the development of the disease [4].

The recommended gold standard for diagnosing endometriosis is laparoscopy, an invasive surgical procedure, with or without histologic verification [1,5]. If a histologic examination is performed, then the presence of extrauterine lesions consisting of endometrial stroma, glands, or hemosiderin macrophages, alone or in combination, confirms the diagnosis. Surgical therapy aims to remove endometrial tissue implants and adhesions, slow disease progression, restore normal architecture, and provide temporary relief. However, many professional societies endorse the medical treatment of patients’ symptoms prior to obtaining a final surgical diagnosis [6-8].

Various medical protocols have been suggested for the management and treatment of endometriosis. The currently available hormonal treatments include oral contraceptives (estrogen-progestogen preparations), progestogen preparations (containing progesterone derivatives), androgenic hormones (danazol), and gonadotropin-releasing hormone (GnRH) agonists and antagonists [6]. Oral contraceptive pills (OCPs) are intended to reduce the levels of follicle-stimulating hormone (FSH) and thus stabilize the endometrium, leading to the reduction of pain-related symptoms. In a prospective cohort study, Vlahos et al. [9] examined the efficacy of the use of both cyclic and continuous combined oral contraceptive pills (OCPs) to improve endometriosis-related pain symptoms following surgical treatment. OCPs, when used continuously, appeared to reduce the recurrence rate of nonmenstrual pelvic pain, endometrioma, and dysmenorrhea, but not dyspareunia, when compared to cyclic OCPs [9]. The use of progestins has been shown to alleviate pelvic discomfort associated with endometriosis. Progestins are generally less costly than other medicines and can be used as tablets, injections, or intrauterine contraceptives that include levonorgestrel. Progesterone derivatives function by reducing the levels of GnRH and subsequently the levels of FSH, luteinizing hormone (LH), and estrogen. The suggested therapy should be used for at least six months [7]. The most prevalent adverse effects of progestin treatment are progressive uterine bleeding, increased weight, retention of fluids, breast discomfort, headaches, nausea, and mood changes, often depression [8]. Danazol, a derivative of the hormone testosterone, is also used in the treatment of endometriosis. It decreases the secretion of FSH and LH by the pituitary gland through the inhibition of GnRH secretion, and the current protocol recommends commencing therapy on day 2 of the menstrual cycle and continuing it long term for at least 6-9 months. The adverse effects include breast reduction, weight gain, oily skin and acne, and fluid retention [7].

Oral contraceptives, synthetic progestins, and danazol were used to treat endometriosis before GnRH agonists were developed. The analogs of GnRH generally include artificial derivatives of the hypothalamic native peptide, coupled with modifications of their chemical structure that generate differences in biological action. Because GnRH causes anovulation and amenorrhea, GnRH agonists are used to treat endometriosis. The same cyclical hormonal influences affect ectopic endometrial implants as they do normal endometrium. Numerous GnRH agonists are available for therapeutic use, and all of them work through the same mechanism. Initially, gonadotropin and gonadal steroid production is stimulated and then inhibited by the downregulation of the pituitary GnRH receptors (GnRHRs). GnRH agonists have proven to be very efficient in treating gonadal steroid-driven disorders and lead to few adverse effects, but long-term usage in women may lead to calcium loss from bone as a result of hypoestrogenism [7,10]. Pharmacologic doses of GnRH agonists administered for six months have been found to produce amenorrhea and anovulation in numerous controlled, randomized clinical studies [11].

Elagolix is an oral, nonpeptide GnRH antagonist, and recent studies showed that it is efficient in controlling both nonmenstrual pelvic pain and dysmenorrhea secondary to endometriosis, with an acceptable safety profile at a dosage of 150 mg/day that produces partial estrogen suppression [12-14]. A phase 2 study has shown that elagolix can lead to nearly full estrogen suppression [13]. Consistent with the mechanism of action, it can result in hypoestrogenic effects, such as changes in bone mineral density and lipid levels, as well as hot flashes.

The purpose of this review is to present a summary of GnRH and GnRH receptors and their role in the treatment of endometriosis. The innovative use of GnRH analogs and antagonists in the treatment of endometriosis is also discussed.

## Review

GnRH and GnRH receptors

GnRH was first isolated from porcine [15] and ovine [16] hypothalamus extracts. In humans, a single copy of the GnRH gene, which consists of four exons and three introns, is found on the short arm of chromosome 8. The GnRH receptor (GnRHR) belongs to the G-protein-coupled receptor family. GnRH binds to this high-affinity seven-transmembrane receptor located on the surface of anterior pituitary gonadotropic cells. Different signaling cascades and transcriptional mechanisms, which also depend on variations in the frequency of GnRH pulse, are activated to stimulate the synthesis and release of FSH and LH. Although GnRH pulse frequency variation may explain some differences in FSH and LH regulation, additional variables such as activin, inhibin, and sex hormones also contribute to the synthesis of gonadotropin. FSH and LH regulate gonadal ontogenesis and steroidogenesis in both sexes [17-19].

GnRH was initially considered to have a single structure in all animal and human species, with its main and fundamental physiological role being the management of LH and FSH release and their biosynthesis in the anterior hypophysis. However, other forms of this peptide have been found, and 30 structurally distinct GnRH forms have been identified so far. Fifteen structural variations of the GnRH molecule occur in vertebrates. Among the 15 variations that have been identified in invertebrates, nine distinct GnRHs have been detected in protochordates, which are closely related to vertebrates, and six more GnRH sequences were established in other invertebrates. GnRH-like peptides found in invertebrates that developed before tunicates (echinoderms, mollusks, and annelids) contain two extra amino acid residues in positions 2 and 3; however, the essential amino acids are conserved in these peptides. The first protostomic invertebrate GnRH sequence was found in octopus, and this sequence and similar invertebrate sequences constitute a distinct group of GnRH peptides of 11 or 12 amino acid residues in length. These sequences are distinguished from GnRHs by the insertion of two amino acids after position 1 and by the modification of the residue of the proline at position 9 of the sequence [20,21]. After the discovery of the GnRH regulatory function in reproduction [22,23], GnRHs were found to also be able to act in a paracrine manner in the placenta and gonads. In addition, GnRHs were discovered to act in an autocrine manner in immune cells and neurons and to play a neurotransmitter/neuromodulatory role in both the peripheral and the central nervous system (CNS) (e.g., in sympathetic ganglions and the midbrain). All these activities can be performed by a single form of GnRH, but at least two or even three distinct GnRHs are widely recognized in most vertebrates [22].

GnRH I was the first GnRH isoform found in the mammalian brain (pGlu-His-Trp-Ser-Tyr-Gly-Leu-Arg-Pro-Gly-NH2); GnRH II (His5, Trp7, and Tyr8) was found to be highly prevalent in vertebrate animals, such as mammals; and a third isoform was detected in fish (salmon). The GnRH I isoform is released by the hypothalamus, but it can be present in various tissues and organs of the body, where it exerts neuroendocrine, autocrine, and paracrine actions [17,20]. The GnRH II isoform varies from GnRH I in positions 5, 7, and 8 by three amino acid residues (His, Trp, and Tyr). Although GnRH II can stimulate the production of gonadotropin, it has been shown to be significantly less effective than GnRH I (approximately only 2% of GnRH I’s effectiveness). The GnRH II receptor is located in suprachiasmatic, periventricular, supraoptic, and medially based hypothalamus nuclei, while the conventional GnRH I exhibits a wide expression pattern. GnRH II is also found in various peripheral tissues, including the ovary, endometrium, and placenta [21]. The GnRH II-specific receptor referred to as the type II receptor was isolated from nonhuman primates, and it contains several phosphorylation sites on its intracellular tail. In humans, the gene encoding GnRHR II has a sequence for an extracellular loop, and it is generally recognized that a functional full-length GnRHR type II protein is not fully expressed in humans. Another isoform, known as GnRH III, has been isolated from sea lamprey (*Petromyzon marinus*); it has a 60% homology with GnRH I with four different amino acids. This peptide has been shown to be a significant part of reproduction in jawless fish (gametogenesis and steroidogenesis) [24].

Physiology of GnRH receptor

GnRH, first identified in 1984, is a decapeptide and is part of the hypothalamic-pituitary-gonadal axis; it is produced in the arcuate nucleus of the hypothalamus and delivered to the anterior pituitary gland [25-27]. GnRH has been shown to exhibit additional actions because of its vast distribution in peripheral organs and tissues and in the central nervous system (CNS), while it is the single major regulator of the hypothalamic-pituitary-gonadal reproductive axis [28]. The potential control of the GnRH/GnRHR system over the hippocampus has increased interest in the influence of the GnRH decapeptide and its analogs on neurogenesis and neural functions. GnRH has been shown to be reduced in the hypothalamus of aged mice, implying that restoring normal GnRH levels might attenuate brain and systemic aging processes [29].

In summary, the GnRH/GnRHR system in the brain represents a new and highly intriguing research field because it mediates numerous activities that may be associated with neuroprotection, neurogenesis, cognition, and sexual behavior, as part of the complex regulation of reproductive processes.

The very low levels of implantation and pregnancy success, which are 5.26% and 15.82%, respectively, are one of the most disappointing elements of today’s assisted reproductive technology (ART) [30,31]. Intensive research on endometrial receptivity is expected to be an essential stage in the embryonic implantation process for patients with recurrent failures in implantation or recurrent miscarriages. The endometrium of females undergoes extensive proliferation and angiogenesis for an easier renewal of the inner linings following each menstrual cycle to enable successful implantation. For a successful reproduction, this extraordinary endometrial regeneration capacity is critically required. The regeneration potential of the endometrium is attributable to endometrial stem cells that may not have been released throughout each menstrual cycle (basal layer). Lucas et al. (2016) [32] have shown that the repetitive loss of pregnancy is closely related to a relative absence of endometrial stem cells. Many studies have shown that GnRH acts as a negative regulator for cancer cell development in the breast, endometrium, and prostate. Furthermore, other studies showed that GnRH can induce apoptosis and reduce the proliferation of endometrial cells in eutopic and ectopic endometrium. It might, thus, be assumed that long-term exposure to GnRH damages endometrial stem cells directly and decreases the favorable results of in vitro fertilization (IVF) procedures in pregnancy. It could be hypothesized that GnRH decreases several favorable activities of endometrial stem cells in vivo and in vitro, such as proliferative and migratory potential. More importantly, GnRH has been shown to substantially suppress different activities of tissue-derived human adipose stem cells, suggesting that it may serve as a global inhibitor of stem cells in many tissue types.

The most prevalent gynecological malignancy in the world is endometrial cancer (EC) [33]. This type of cancer is considered a malignant tumor of the epithelial cells of the endometrium, and it is sporadic in about 90% of cases. The treatment of advanced disease or recurrence in women includes chemotherapy, endocrine therapy, or new therapeutic strategies (e.g., angiogenesis, HER2, epidermal growth factor receptor (EGFR), and mammalian target of rapamycin (mTOR) inhibitors). Surgery and chemotherapy or even antiangiogenic medicines (bevacizumab) are the chosen option for advanced or initial stages with a higher chance of recurrence. However, EC and epithelial ovarian cancer (EOC) patients often relapse despite improved responses to traditional and recently established treatment strategies. In the last decades, there has been considerable research on the expression pattern and function of the GnRH/GnRHR bioregulation system in female reproductive cancers. GnRHR is highly expressed in ovarian and endometrial cell lines and in approximately 80% of the developed primary tumors. The activation of GnRHRs that are expressed locally inhibits cell proliferation and metastatic behavior, indicating that this system functions as a tumor growth inhibitor. These findings support the notion that these receptors could be a molecular target for new GnRH analog-based treatment strategies for gynecologic cancers [34].

GnRH agonists are claimed to exert major antiproliferative effects that occur in endometrial and ovarian cancer, as well as in primary cell cultures. GnRH antagonists also suppress the growth of carcinoma cells in the endometrium and ovary tissues and confirm that drugs behave similarly to the GnRHR agonists in reproductive tissue tumors. In particular, the possible role of GnRH II, studied in various mammalian tissues including reproductive system tissues, is to limit tumor formation. The natural analogs of GnRH II were found to have significant antiproliferative actions on human ovarian and endometrial cells by activating the classical (type I) GnRHR. Despite the consistent in vivo and in vitro verification of the direct antitumor actions of GnRH analogs, the use of antitumor compounds in ovarian and endometrial cancer is bounded to cases where the main aim is to prevent the blocking of gonadal estrogen separation, e.g., in tumors with dependency on estrogen.

Currently, the treatment of various tumor types involves more focus on alternative therapies. GnRH derivative peptides may be used as carriers or target moieties for the specific delivery of chemotherapeutic medications based on their interaction with GnRHRs produced by cancer cells.

Clinical use of GnRH in the management of endometriosis

The first-line medical therapy (combined oral contraceptive pills or progestogens) for endometriosis is effective for approximately two-thirds of individuals experiencing endometriosis-related pain [35]. GnRH agonists are new types of medication, leading to significant decreases in testosterone and estrogen blood levels for the duration of treatment. The earliest GnRH agonists licensed for use in the United States were goserelin acetate (Zoladex) and nafarelin acetate (Synarel), as well as leuprolide acetate (Lupron), which have shown outstanding effects. Advanced prostate cancer, precocious puberty, and endometriosis are approved indications for these medicines depending upon the exact substance. These and other GnRH experimental agonists have been also studied in a wide variety of different diseases, involving uterine fibroids, ovarian polycystic disease, fibrocystic breast, benign bowel, and prostatic hypertrophy. The GnRH agonists are one of the most remarkable developments in hormone therapy in recent decades. Synthetic GnRH agonists were developed, based upon the observation that the naturalized GnRH peptide is rapidly degraded in the circulation, with the degradation occurring at the Gly residue in position 6; additionally, the COOH terminus of the GnRH decapeptide is essential for receptor binding, while the NH2 terminal end is required not only for receptor binding but also for receptor stimulation [20]. Hence, GnRH agonists have been created by substituting Gly6 with a D-amino acid, resulting in a longer plasma half-life than the original hormone; furthermore, some of these compounds contain a deletion of Gly10-amide with the inclusion of an ethylamide residue to Pro9, resulting in a higher affinity for GnRHRs [19,36,37]. Drugs such as goserelin triptorelin, buserelin, and leuprolide are the most often utilized GnRH agonists in clinical studies; however, it was soon revealed that GnRH agonists, after the first stimulating impact, known as the “flare effect,” paradoxically result in the gradual and persistent decrease in the gonadotropin production. The second impact, called downregulation, can be detected after approximately 10 days. Since this phase is reversible when medication has been stopped, it can be sustained if GnRH agonists are used for a long time. GnRH agonists can also be administered pulsatively using a pump to stimulate the long-term production of gonadotropin, e.g., for inducing puberty [38,39].

Clinical studies have been conducted on nafarelin, a GnRH agonist. Specifically, in a randomized clinical trial, 104 patients received 400 μg of nafarelin per day and 63 received 600 mg of danazol per day [40]. Pelvic discomfort was almost entirely gone after two months of GnRH agonist or danazol treatment. The average laparoscopic score, which indicates implant size reduction, dropped from 20.4 to 11.7. After completion, the pregnancy rate for individuals who were trying to conceive was 39%.

Similar suppressing effects on endometriosis are observed with other GnRH agonists. Many reports have been documented on buserelin stating its beneficial effects at a dose of a minimum of 0.2 mg/day subcutaneously or a maximum of 12 mg/day intranasally for six months or longer [41].

Additional formulations studied in controlled clinical trials are goserelin as a subcutaneous repository and leuprolide acetate as an intramuscular presence, with comparable side effects [42]. The adverse effects of GnRH agonist treatment are restricted to those due to hypoestrogenism, involving headache, hot flashes, vaginal dryness, reduced libido, mood alterations, and a reversible bone mineral density loss by 2% or 8% after six months [38,39,43]. Their effectiveness in releasing the symptoms of endometriosis is similar to that of nafarelin.

All GnRH agonists have been proven to inhibit endometriosis in the same way. Infertile women treated with GnRH agonists for endometriosis-related infertility had a pregnancy rate comparable to those treated with danazol or pregestational drugs. After stopping GnRH agonist therapy, ovulatory cycles generally revert to normal in one to three cycles. Approximately 15% of individuals have recurring diseases and require additional treatment within 12 months following a six-month-long process with GnRH. During the 12-month follow-up period, symptom scores increased, but they remained below the baseline pain levels [44]. The earliest studies using GnRH agonists for endometriosis examined the effects of the duration (months) of therapy based upon the danazol regimen, which was the first medication for endometriosis authorized by the US Food and Drug Administration. Women receiving GnRH agonists reported alleviation of symptoms 4-8 weeks after starting therapy [44].

Endometriosis symptoms often recur 9-12 months after medical treatment has ended. As a result, the effect of a recurrent short course of GnRH agonist therapy has been investigated [43]. Tahara et al. (2000) [43] investigated the decrease in endometriosis symptoms and differences in adverse effects in two groups of patients who were randomized to receive either full-dose nafarelin treatment (200 μg twice daily) for 24 weeks (n = 7) or full-dose nafarelin treatment for four weeks followed by half-dose nafarelin treatment (200 μg/day) for 20 weeks (n = 8). The authors concluded that there was similar improvement in symptoms in both groups; however, adverse effects were markedly reduced with half-dose administration.

GnRH antagonists, which have been commercially available since 1999, have been utilized to avoid premature LH surges of the ovarian in vitro or intracytoplasmic sperm injection stimulation in the controlled stimulation. In the context of the increased evidence, GnRH antagonists are safe and effective, allowing the flexible treatment of an extensive range of patient groups [45,46]. GnRH antagonists include first-line controlled poor responders, ovarian stimulation, and women with polycystic ovary syndrome (PCOS). The GnRH antagonists represent a feasible alternative to the long-term agonists, which allow a shorter period of treatment with fewer injections and no detrimental effects. This leads to a considerably decreased need for gonadotropins, which can lead to better compliance. GnRH antagonists were created with the goal of obtaining drugs that would cause the blockage of the pituitary-gonadal axis without causing the unwanted flare effect. GnRH adversaries affect reproductive functions directly. They interfere with and occupy hypophysis GnRH receptors, limiting access to their necessary place of identification by endogenous GnRHs and exogenously managed agonists [47]. Unlike GnRH agonists, GnRH antagonists instantly inhibit GnRH receptors by competition over pituitary gonadotropins and offer flexibility in the degree of pituitary-gonadal suppression.

The strongest total inhibitory action and receptor binding affinity were shown by cetrorelix, ganirelix, abarelix, and all GnRH antagonists; however, abarelix was shown to cause immediate-onset systemic allergic responses. Ganirelix and cetrorelix have been shown to be notably effective in adjusting ovarian stimulation methods for the avoidance of an early LH surge in vitro fertilization technology [46,48-50]. Recent studies have focused on the results of oral GnRH antagonists linzagolix and relugolix in the management of endometriosis-related symptoms [50,51]. These two studies showed that the GnRH antagonists suppress ovarian function in a dose-dependent manner, allowing the modulation of the E2 levels according to the threshold hypothesis while minimizing the side effects secondary to hypoestrogenism.

It becomes apparent that there are many treatment options, and it is the responsibility of the healthcare providers to offer women a comprehensive overview of the efficiency and side effects of all available therapies, as appropriate counseling is of extreme importance. The optimal treatment should be tailored to each patient according to endometriosis-related symptoms and the phenotype of the disease. In addition, further research is needed to investigate innovations in treatment options, such as the use of the exogenous administration of GnRH II or GnRH I receptor antagonists to improve access to quality care.

## Conclusions

Endometriosis is an enigmatic disease that develops due to the implantation of endometrial tissue outside the uterus. The disease shows multiple severe symptoms including abnormal bleeding, inflammation, chronic pelvic discomfort, and infertility. Therapeutic strategies are under continuous development and currently include progestin, elagolix, letrozole, anastrozole, and exemestane with estrogen-suppressing medications. In vitro studies in endometrial cell lines have enhanced the knowledge about the role of the GnRH/GnRHR system in the disorder. In addition to laparoscopy, medical therapies using GnRH agonists, leuprolide, and goserelin can be helpful in the treatment of endometriosis. However, these treatment approaches have some adverse effects, unlike GnRH antagonist, which inhibits gonadotropin secretion without inducing flare effect while competing with natural GnRH. In addition to the therapeutic use of GnRH analogs, the regulation of GnRH pulsatile release provides new research prospects for the diagnosis and treatment of female reproductive system disorders and their resulting complications, such as tumor development and iatrogenic diseases.
